# High content live cell imaging for the discovery of new antimalarial marine natural products

**DOI:** 10.1186/1471-2334-12-1

**Published:** 2012-01-03

**Authors:** Serena Cervantes, Paige E Stout, Jacques Prudhomme, Sebastian Engel, Matthew Bruton, Michael Cervantes, David Carter, Young Tae-Chang, Mark E Hay, William Aalbersberg, Julia Kubanek, Karine G Le Roch

**Affiliations:** 1 Department of Cell Biology and Neuroscience, University of California Riverside, Riverside, CA 92521, USA; 2 School of Chemistry and Biochemistry, Georgia Institute of Technology, Atlanta, GA 30332, USA; 3 School of Biology, Georgia Institute of Technology, Atlanta, GA 30332, USA; 4 Institute for Integrative Genome Biology, University of California, Riverside, CA 92521, USA; 5 Laboratory of Bioimaging Probe Development, Singapore Bioimaging Consortium, Biopolis 138667, Singapore; 6 Agency for Science, Technology and Research (A*STAR), Biopolis 138667, Singapore; 7 Institute of Applied Sciences, University of the South Pacific, Suva, Fiji

**Keywords:** *Plasmodium falciparum*, Drug screening, Natural products, Antimalarial, High-throughput screening

## Abstract

**Background:**

The human malaria parasite remains a burden in developing nations. It is responsible for up to one million deaths a year, a number that could rise due to increasing multi-drug resistance to all antimalarial drugs currently available. Therefore, there is an urgent need for the discovery of new drug therapies. Recently, our laboratory developed a simple one-step fluorescence-based live cell-imaging assay to integrate the complex biology of the human malaria parasite into drug discovery. Here we used our newly developed live cell-imaging platform to discover novel marine natural products and their cellular phenotypic effects against the most lethal malaria parasite, *Plasmodium falciparum*.

**Methods:**

A high content live cell imaging platform was used to screen marine extracts effects on malaria. Parasites were grown *in vitro *in the presence of extracts, stained with RNA sensitive dye, and imaged at timed intervals with the BD Pathway HT automated confocal microscope.

**Results:**

Image analysis validated our new methodology at a larger scale level and revealed potential antimalarial activity of selected extracts with a minimal cytotoxic effect on host red blood cells. To further validate our assay, we investigated parasite's phenotypes when incubated with the purified bioactive natural product bromophycolide A. We show that bromophycolide A has a strong and specific morphological effect on parasites, similar to the ones observed from the initial extracts.

**Conclusion:**

Collectively, our results show that high-content live cell-imaging (HCLCI) can be used to screen chemical libraries and identify parasite specific inhibitors with limited host cytotoxic effects. All together we provide new leads for the discovery of novel antimalarials.

## Background

Malaria remains a major public health issue in developing nations. In 2006, the World Health Organization reported approximately 250 million cases of malaria, which caused 1 million deaths a year [1]. Despite such a high number of deaths per year, malaria is a curable disease and traditional medicinal plants have been used for treatment since antiquity. Native Peruvians used the bark of the *Cinchona succirubra *(Rubiaceae) tree for centuries before quinine was isolated from it in 1820 [2]. Its semi-synthetic derived compound, chloroquine (CQ), became the prophylactic treatment for malaria in 1947 and was the most effective treatment until CQ-resistant strains appeared in 1957. In 1972, a new natural product, artemisinin, was isolated from *Artemisia annua*, a plant used in traditional Chinese medicine for over 2000 years [3]. Artemisinin-based combination therapies (ACTs) are currently our last resort in combating malaria infection. Unfortunately, the first ACT-resistant strains appeared in Cambodia in 2009 and hasten the need for new antimalarials [4]. In the long history of drug discovery against the human malaria parasite it is clear that natural products have outlived many synthetic drugs and remain a valuable resource in identifying efficient and long lasting novel antimalarials.

One successful approach in discovering new chemical and natural therapeutic agents against the malaria parasite is based on high-throughput screening (HTS) on the whole organism. Large collections of small molecule libraries can be tested directly against parasite growth in culture [5-8]. Traditionally, the [3^H^]hypoxanthine incorporation assay was the gold standard to determine, *in vitro*, the drug susceptibility of the malaria parasite [9]. This method has now been largely replaced by less hazardous, cost and labor efficient DNA dye intercalation assays (SYBR Green I [10,11], Pico green [12], 4', 6-diaminino-2-phenylindole (DAPI) [13]), assays using quantum dots labeling late stage infected erythrocytes [14], and assays using parasites that stably express cytoplasmic firefly luciferase [15,16]. While the various DNA dye assays are capable of quantifying parasite growth, they are limited to testing a simple survival count and do not efficiently detect the effect of drug treatment at the morphological level or provide information of a potential drug's cytotoxicity. Current screenings with parasite strains expressing green fluorescence protein (GFP) have facilitated the observation of the dynamic behaviors of parasite phenotype in a real-time manner. However, these techniques require the use of a modified cell line for all screening purposes.

Recently, we developed a semi-automated RNA fluorescence-based high-content live cell-imaging (HCLCI) assay that has multiple advantages [17]. It is a fast, simple and a one-step fluorescence-based assay that can be used with any type of *Plasmodium *laboratory and field isolate strains. It can detect a very low number of live parasites, their morphological stages and their transcriptional activities. When high-quality bioimaging microscopes and image- analysis tools are combined, these screening platforms can facilitate the detection of cytotoxicity or cellular phenotypic changes in the parasite population and its host cell. Therefore, this assay can potentially lead to the discovery of novel drugs with novel modes of action and a hint toward the identification of their cellular targets.

In this study, we have used our new live cell confocal imaging platform to identify new parasite inhibitors from crude extracts and their specific morphological effects on *Plasmodium falciparum*. Our natural product library was first extracted from a rich source of various sessile marine organisms (e.g., red macroalgae, benthic cyanobacteria, cnidarians, and sponges). Sessile marine organisms are exposed to numerous pathogens and predators in seawater and have developed effective natural chemical defenses [18,19]. Their natural products have been shown to have biological activity against a wide range of human cancer cell lines and several infectious agents [20,21].

To screen our marine natural product library for antimalarial activity, we first employed a standard SYBR Green I based assay. Extracts that inhibited parasite growth at low concentrations were then subjected to a secondary HCLCI screen to examine parasite morphologies and the toxicity of natural product treatment on host red blood cells. Collectively, this live cell-imaging assay improves the spatial and temporal resolution of the malaria parasite screening assay, and provides valuable information about the health of host cells under drug treatment. Potent novel antimalarials were identified and they produced phenotypic irregularities in the malaria parasite, but had minimal cytotoxic effects on host red blood cells. Further validation of our methodology was achieved using the newly purified natural compound bromophycolide A. All together our data demonstrate that this streamlined assay can be used to discover novel and efficient antimalarials.

## Methods

### Parasite culture

3D7, HB3 and Dd2 *P. falciparum *malaria parasites (MRA-102, 155, 156, MR4, ATCC^® ^Manassas, Virginia) were cultured in human type O + erythrocytes in complete medium (RPMI 1640 w/L-Glutamine, w/o Phenol Red (Gibco), 0.043 mg/mL Gentamicin (Gibco), 0.014 mg/mL Hypoxantine (Acros), 38.5 mM HEPES (Promega), 0.18% Sodium Bicarbonate (Gibco), 0.2% Glucose (MP Biomedical), 2.6 mM NaOH (Sigma), 0.20% Albumax (Gibco), 5% human serum) as previously described [22]. Cultures were maintained in 25-cm2 flasks (Corning) at a volume of 10 ml and were gassed for 30 s with an environment of 3% CO2, 1% O2, and 96% N2, then incubated at 37°C. Synchronization of culture was achieved through sorbitol lysis of mature stage using 5% sorbitol (Fisher) fine-tuned by another lysis 8 h later (27).

### Marine organism extracts

Each marine organism was exhaustively extracted with methanol, and the extracts were combined, filtered, and reduced *in vacuo*. The crude extract was separated with HP20ss resin into four fractions, eluting with (1) 1:1 methanol: water, (2) 4:1 methanol: water, (3) 100% methanol, and (4) 100% acetone.

### Plasmodium falciparum SYBR Green I-based fluorescence growth inhibition assay

Extracts were diluted into three different concentrations, 100, 33, and 11 μg/ml, by the Biomek FXP Laboratory Automation Workstation (Beckman Coulter, Inc., Brea, CA) into clear bottom 96-well plates (Costar #3904). *P. falciparum *cultures were added at a 2.5% hematocrit with a 1% parasitemia to 96-well plates and incubated for 72 h at 37°C. Plates were frozen at -80°C overnight. Lysis buffer (20 mM Tris-HCl, 5 mM EDTA, 0.008% saponin, 0.08% Triton X-100, and 0.2 μl/ml SYBR Green I dye (Invitrogen #S7585)) was added to thawed plates and incubated at 37°C. Microtiter plate was read using the Spectra Max Gemini EM reader (Molecular Devices). Data were analyzed with SoftMax Pro v5 (Molecular Devices Software, Inc., Sunnyvale, CA). The formula z' = 1 - (3SD+ + 3SD-)/|Ave + - Ave-| was used to calculate the Z' value. Where SD + represents the positive control standard deviation, SD- the negative control standard deviation, Ave + the mean value of the positive control, and Ave- the mean value of the negative control.

### Bacterial growth inhibition assay

Methicillin-resistant *Staphylococcus aureus *(MRSA) and vancomycin-resistant *Enterococcus faecium *(VREF) as test pathogens were used to perform antibacterial assays. As previously described [20], these bacteria were grown in GYT media (1 g glucose, 2.5 g yeast extract, 5.0 g tryptone, 1 L H2O) overnight at 37°C. Cultures were diluted to an optical density of 0.04-0.06, diluted 10-fold, and then 250 μg/ml of extracts were added to microtiter plates. Extracts were serially diluted for a total of 8 dilutions, and the plates were incubated for 16-18 h at 37°C. The optical density was then measured at 600 nm using a Molecular Devices Emax microplate reader and the minimum inhibitory concentration (MIC) was calculated for each compound using the analysis program SOFTmax PRO.

### Fungal growth inhibition assay

As previously described [20] amphotericin B-resistant *Candida albicans *was grown overnight at 30°C in RPMI media (Invitrogen). The indicator Alamar Blue 100 × (TREK Diagnostic Systems) was added to the cell suspension at 1 × 104 cells/mL, and then cells were added to microtiter plates. Extracts were added to 96-well plates at 250 μg/ml, serially diluted for a total of 8 dilutions, and incubated for 12-15 h at 37°C. The visual assessment of colorimetric changes was done using a BioTek model ELx800 microplate reader to detect the altered oxidation state of Alamar Blue. Plotting the absorbance versus compound concentration indicated cell proliferation.

### High-content live cell imaging assay, Montage analysis

Samples were prepared in optical bottom 96-well assay plates (Costar #3614, Corning, NY). Parasite cultures were diluted to 0.025% hematocrit with a 6% parasitemia in complete medium and 240 μl was added to each well. RNA-probe 132A was incubated with samples for 30 min at 37°C, in the dark at a final concentration of 5 μM. The BD Pathway HT (BD Biosciences Bioimaging, Rockville, MD) incubation chamber was set at 37°C and 5% CO2. Individual wells were fluoresced by Semrock (Rochester, NY) Texas Red BrightLine filter sets. A 3 × 3 tiling composed montage images to examine approximately 1500 red blood cells.

### IC_80 _phenotypic assay

Parasite cultures were synchronized using 5% sorbitol as previously described [23]. Cultures were then diluted to 1.67% hematocrit with a 6% parasitemia for ring stage and 3% mature stage in complete medium to reach a final volume of 240 μl. Treatment with extracts or bromophycolide A at their respective IC_80 _values were added at 0, 8, and 16 h, and image analysis occurred at 24 h.

## Results and Discussion

### Crude extract library preparation

**Figure 1:**
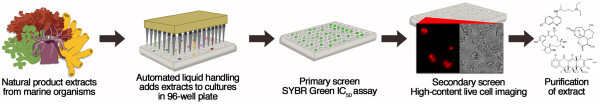
**A schematic diagram of natural products antimalarial screen**. Marine organisms were collected from the reef off the Fijian Islands. Extracts were aliquoted into 96-well microtiter plates and cultures were subsequently added. A SYBR Green I based assay was used as a primary screen of 2685 extracts. Extracts that inhibited *P. falciparum *growth were subjected to a high-content live cell imaging secondary screen.

To investigate novel antimalarial compounds, we prepared a library of crude extracts derived from various marine organisms including red, green, and brown macroalgae, cyanobacteria, cnidarians, and sponges collected from the Fiji Islands. Figure 1, a schematic flow chart, illustrates the key steps in our screen. Extracts were first separated by reversed-phased column chromatography and four fractions ranging in compound polarity were collected for each individual organism. All fractions were tested for antimalarial activity.

### Primary bioactivity screen of natural products

Our extract library was screened for antimalarial activity using a standard high-throughput (HT) SYBR Green I based assay. We employed the Biomek automated liquid handling system to aliquot extracts into three different concentrations (100, 33, and 11 μg/ml) in 96-well plates. *P. falciparum *cultures were added and incubated for 72 h before being subjected to a HT assay to evaluate parasite growth by relative fluorescence intensity of the nucleic acid dye SYBR Green I. To validate the quality of our HT SYBR Green I assay we calculated its Z-factor, which is a high-throughput assay quality assessment. An ideal Z-factor is 1.0, and below a 0.5 is a marginal assay. Our primary screen Z-factor value was 0.669, indicating an excellent primary assay to determine parasite growth inhibition based on relative fluorescence units. From the initial library of 2685 marine extracts examined, 27 extracts inhibited parasite growth below 11 μg/ml. With approximately 1% of extracts found to be potent inhibitors and based on our Z-factor score, our primary assay is efficient and effective for screening our library and marine organisms are a novel source for new antimalarial therapies. While this assay gives us an indication of the parasite survival, it does not provide any indication of the drug effects on the parasite morphology and potential toxicity on host red blood cells (RBCs).

### Secondary high-content live cell imaging screen

To further validate antimalarial activity, extracts that inhibited parasite growth below 11 μg/ml were selected and subjected to a secondary HCLCI screen. By using this technique, we could easily visualize our live parasites in host red blood cells. For this secondary screen, we used the Bioscience Pathway HT, an inverted confocal microscope with a motionless stage in a climate-controlled environment together with a fluorescent RNA selective probe (132A). This RNA probe has previously been shown to permeate infected erythrocytes without additional lysis buffers or detergents [17]. When compared to DNA binding dyes (DAPI, Hoechst) used in current HT antimalarial screens, our selected RNA probe diffuses throughout the cytoplasm and provides detailed live cell staining of *P. falciparum*. Using this methodology we were able to evaluate the parasitemia, morphology and transcriptional activity under drug treatment. In addition to that information, the transmitted light images provide valuable information on potential toxic effects of the extracts on host RBC morphology. For example, it has been previously observed that erythrocytes crenate and transform into echinocytes when cell membranes are perturbed [24]. A characteristic of an altered RBC membrane is the formation of small knoblike spicules on the membrane surface that causes the cell to transform from a biconcave disk, discocyte, into a sea urchin-like shape, echinocyte. There are 3 stages of echinocyte transformation. In stage 1 RBCs start to have an irregular contour disk shape, stage 2 cells are still flat with spicules, and stage 3 cells have an even distribution of 10-30 spicules on the surface [25]. Transmitted light images provide insight whether extracts compromise host RBC integrity and therefore parasite survival, or if the extracts specifically target parasites. Cytotoxic effects of marine extracts on RBC were evaluated by the degree and amount of echinocyte transformation relative to untreated cultures. Extracts that caused cultures to have a 50% increase of stage 3 echinocytes, when compared to untreated cultures, were considered toxic.

From the secondary screen, 10 of these extracts were observed to have minimal echinocyte transformation when compared to untreated parasite culture (data not shown). This second set of selected extracts most likely has a specific antimalarial effect with limited toxicity on RBC integrity.

### Determination of antimalarial specificity of our selected extracts

**Figure 2:**
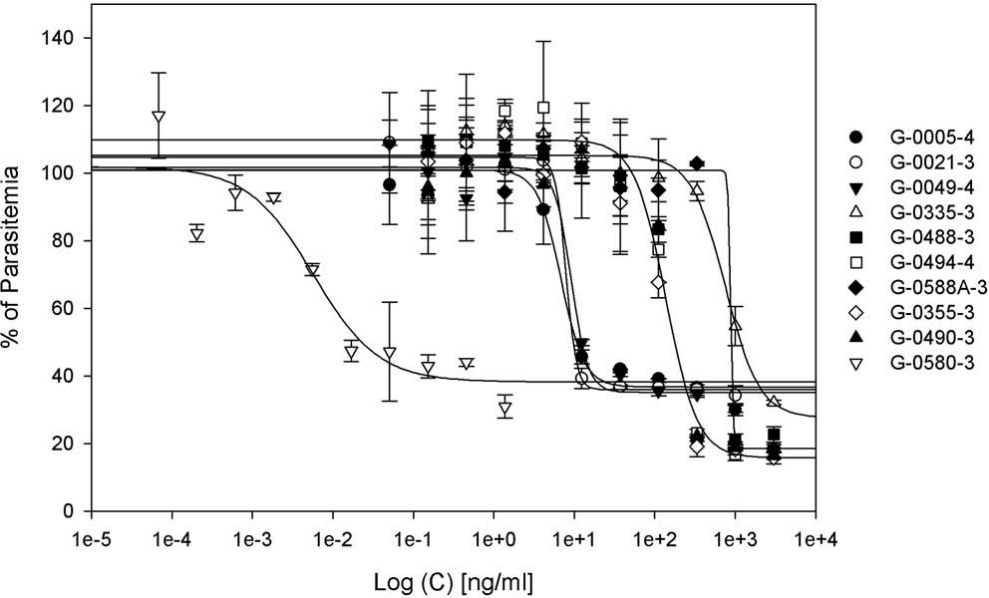
**IC_50 _graph of potent antimalarial extracts**. An inhibitory concentration at 50% growth (IC_50_) graph of extracts inhibiting parasites at concentrations 6.65 ng/ml to 0.85 μg/ml.

To further validate our screening procedure, we determined the IC_50 _values of our selected extracts using the classical SYBR Green I Assay. We found that their exact IC_50 _ranged from 6.65 ng/ml to 0.85 μg/ml (Figure 2). Next we compared the antimalarial IC_50 _values to the IC_50 _values of these extracts against various infectious agents (e.g. methicillin-resistant *Staphylococcus aureus *(MRSA); vancomycin-resistant *Enterococcus faecium (VREF); *and both wild type and amphotericin B-resistant *Candida albicans *(WTCA and ARCA, respectively). MRSA is a pathogenic bacterium that causes skin and soft-tissue infections and is associated with sepsis and necrotizing pneumonia. VREF is another infectious bacterium that commonly causes urinary tract infections and can cause neonatal sepsis and meningitis. *C. albicans *is a fungus that lives in the gut of 80% of the human population, but can be lethal in immunocompromised patients. Most extracts were inactive against WTCA and ARCA. Results show that our selected extracts inhibit growth of the *P. falciparum *3D7 strain at 10-200-fold lower concentrations compared to other infectious agents (Table 1). Overall, our data obtained from the microscopic analysis of parasite and host RBC and other infectious agents indicated that our selected extracts are most active against *P. falciparum*.

**Table 1:** IC_50 _values of seven extracts in *P. falciparum *and other microbes.

	3D7	MRSA	VREF	WTCA	ARCA
G-0005-4	0.680 ± 0.13	NT	16.4	227	156

G-0021-3	0.790 ± 0.17	11.9	15.2	> 250	250

G-0049-4	0.916 ± 0.19	8.5	7.1	221	225

G-0335-3	0.775 ± 0.25	62.5	> 250	> 250	> 250

G-0355-3	0.131 ± 0.02	1.9	69.2	> 250	> 250

G-0488-3	0.119 ± 0.03	0.5	> 250	1.08	1.11

G-0490-3	0.163 ± 0.02	1.26	67.61	> 250	> 250

G-0494-4	0.137 ± 0.02	0.5	0.5	> 250	> 250

G-0580-3	0.0055 ± 0.01	92.26	153.11	> 250	> 250

G-0588A-3	0.847 ± 0.09	39.9	107.5	150	150

Extracts are considered a 'hit' if IC_50 _< 100 μg/mL, and extracts with > 250 μg/mL are considered not active. IC_50 _values for 3D7 *P. falciparum *strain are significantly lower than other microbes (MRSA: methicillin-resistant *Staphylococcus aureus; *VREF: vancomycin- resistant *Enterococcus faecium; *WTCA: wild type *Candida albicans; *ARCA: amphotericin-B-resistant *Candida albicans)*. NT indicates not tested, values for *P. falciparum *3D7 samples are from duplicates, and the values for MRSA, VREF, WTCA, or ARCA screens are from a single experiment. Unit values are in μg/mL.

### Phenotypic analysis of parasites incubated with extracts

**Figure 3:**
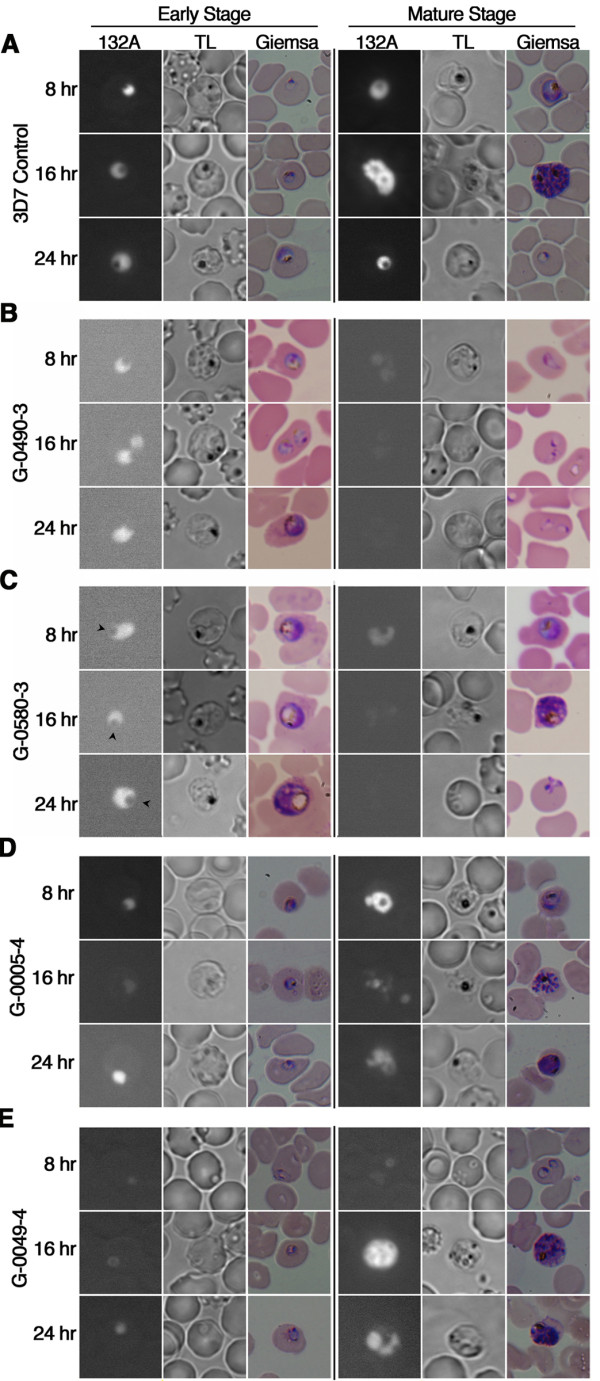
**Morphological analysis of *P. falciparum *with IC_80 _concentrations of extracts**. Infected erythrocytes were synchronized and live parasites were stained with RNA probe 132A. Figure 3a are images of the *P. falciparum *3D7 strain without any drug treatment at the ring and mature stage (control). Figure 3b displays the phenotype of extract G-0490-3, stunted growth of parasites at the trophozoite stage. Figure 3c displays the phenotype of extract G-0580-3, arrowheads indicate the enlarged food vacuoles at the early stages of the cell cycle. Figure 3d and Figure 3e, extract G-0005-4 and G-0049-4 respectively, inhibit parasite life cycle progression, decrease hemozoin, and hinder egression.

**Figure 4:**
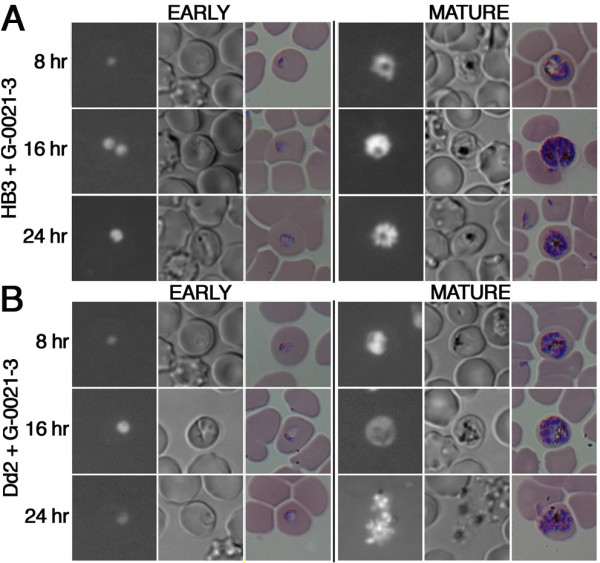
**Morphological analysis of various strains of *P. falciparum***. Treatments with extract G-0021-3 on synchronized parasites at the ring and mature stage. A) chloroquine sensitive strain, HB3; and B) chloroquine resistant strain, Dd2.

To further investigate the potential mode of action of potent extracts, we examined *Plasmodium *morphology within 24 h of treatment. Synchronized parasite cultures were incubated with IC_80 _concentrations of extracts at the early and mature stages of the erythrocytic cycle. As shown in Figure 3a, untreated parasites displayed a robust fluorescent signal with an average of 1000 relative fluorescence units (RFUs) per frame, indicating a high level of RNA and consequently an active transcriptional state. In the transmitted light images, hemozoin, the byproduct of hemoglobin catabolism, can be observed in all stages as a dark pigmented region within the parasites. We also detected a tight and continuous cell cycle progression during our first 24 h of observation. In contrast to this pattern, when treated with sponge extract G-0490-3, parasite growth was inhibited at the late ring and early trophozoite stage (Figure 3b). Parasites treated at the mature stage displayed a decrease of RNA fluorescence signal intensity to an average of 400 RFUs per frame in ring stage parasites. Following our phenotypic observations, it is highly possible that extract G-0490-3 targets a protein or a pathway essential to the early stage of the parasite intraerythrocytic cycle. This phenotype has been observed with *Cinchona *alkaloid treatment, which arrest development of *P. falciparum *at the mature trophozoite stage [3]. While extract G-0490-3 appeared to be ring stage specific, the cyanobacteria extract G-0580-3 produced a different effect on parasite phenotype (Figure 3c). When this extract was added to ring stage parasites, we observed enlarged vacuoles adjacent to hemozoin. When the same extract was added at the mature stage, multiple abnormal vacuoles appeared (16 h) and parasites were unable to egress properly (24 h). Giemsa stained blood smears confirmed the appearance of large vacuoles. This phenotype has been previously observed with inhibitors targeting protease in the parasite food vacuole. Enlarged food vacuoles are observed due to an accumulation of undegraded globin [26]. All together these data suggest that G-0580-3 may target a similar pathway. Similar phenotypes were observed with treatment with samples G-0005-4 (Figure 3d), G-0049-4 (Figure 3e), and G-0021-3 (not shown) and all were extracted from the red macroalga *Callophycus serratus*. To investigate the morphological effects of our selected extracts, we further examined their effects on chloroquine-resistant, Dd2 (Figure 4a), and chloroquine-sensitive, HB3 (Figure 4b) strains. In Dd2, HB3 and the reference 3D7 strain, we observed the almost identical morphological phenotype of stunted hemozoin formation and cell cycle arrest. Collectively, by using the HCLCI assay we can observe phenotypic changes that can be used as a guide to identifying antimalarial properties on drug resistant and sensitive parasite strains.

### Verification of method using initial extracts from purified bioactive compound bromophycolide A

**Figure 5:**
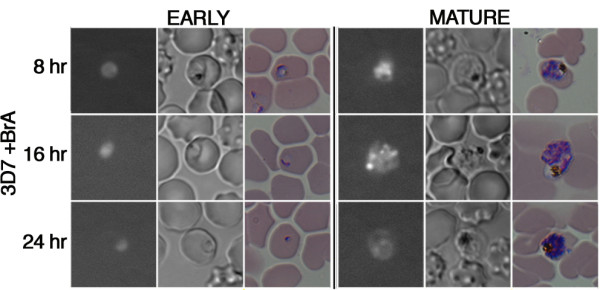
**Morphological analysis of *P. falciparum***. Images of synchronized parasites at the ring and mature stage with bromophycolide A treatment at IC_80 _concentrations. Untreated control sample is shown in Figure 3a.

The purified bioactive compound, (bromophycolides (A-U)), present in G-0005-4, G- 0049-4, and G-0021-3 extracts isolated from *C. serratus *[20,27-29] was shown to inhibit parasite growth at a sub-micromolar range (IC_50 _value of 0.7 μM [28]; IC_80 _value of 2.8 μM). To understand the morphological effects of pure bromophycolide A on *P. falciparum *3D7 cultures, we synchronized parasites and treated cultures at its IC_80 _value at the early and late stage of the parasite erythrocytic cycle (Figure 5). Using our high-content live cell-imaging assay, we detected an immediate arrest of cell cycle progression at every stage tested, a decrease in RNA intensity and an enlargement of the food vacuole in the parasite. Furthermore we observed a hindrance of hemozoin formation from the ring to trophozoite stage. All together, our newly developed screen is able to identify new antimalarials and suggest starting hypotheses for understanding the molecular processes targeted by extracts and their purified bioactive compounds.

Within the past decade significant advancements in high-throughput antimalarial drug screening have efficiently reduced time, labor and costs. However, it remains a challenge to determine the mode of action of active compounds [30]. We propose in this work to use our HCLCI assay to screen large libraries for antimalarial properties. The HCLCI assay is straightforward and allows for semi-automated image analysis of parasite drug resistant and sensitive strains without laborious steps (ex. blood smears, fixation, centrifugation, or aspiration steps). An additional advantage of our assay is the ability to observe cytotoxic effects on host red blood cell. Our method can detect parasite morphological changes to select for specific phenotypes, and further select the extracts for the purification of a single bioactive compound with activity against malaria parasite growth. We observed a distinct phenotype of cell cycle arrest and lack of hemozoin formation when cultures were incubated with extracts from which bromophycolide A was purified and thus pure bromophycolide A as well. These observations indicated that bromophycolide A's potential mode of action may prevent hemozoin formation. Further investigation using a fluorescent coumarin tagged bromophycolide A for sub-cellular localization and molecular target identification studies validated that heme crystallization was disrupted by the natural product bromophycolide A [31].

## Conclusions

In this study we investigated 2685 extracts from sessile marine organisms collected in Fijian waters and successfully identified nine selective extracts that exhibited parasite growth inhibition without compromising RBC membrane integrity. Furthermore we were able to validate that the purified bioactive compound bromophycolide A extracted from the red macroalga *C. serratus*, had similar morphological effects compared to the initial tested crude extracts. Collectively, we demonstrate that by using our newly developed screening we can quickly select a desired phenotype from crude extracts to act as a guide to the purification of a single antimalarial compound. Specific parasite phenotypes can provide insight on how extracts act on the whole organism, thus presenting a starting point to test hypotheses regarding the potential drug's mode of action. In the antimalarial drug field, targeting the parasite at different stages of the life cycle by combinatorial drug treatment is highly recommended to prevent drug resistance. By implementing our screen to classical drug discovery methods, we can expedite the discovery of molecules that can target distinct parasitic stages and lead to new combination drug therapies.

## Abbreviations

HCLCI: high-content live cell-imaging; RBC: red blood cell.

## Competing interests

The authors declare that they have no competing interests.

## Authors' contributions

KLR conceived the study and helped to draft the manuscript. JP and MB prepared samples for the primary screen. SC and DC participated in the design of the HCLCI assay. SC and MC prepared samples and carried out microscopic analysis. JK, MEH and WA provided extracts. SE and EPS established the library and purified extracts. SC drafted the manuscript, and all authors read and approved the final manuscript.

## Pre-publication history

The pre-publication history for this paper can be accessed here:

http://www.biomedcentral.com/1471-2334/12/1/prepub
